# Perineural Invasion of Cranial Nerves in Cutaneous Squamous Cell Carcinoma: Unraveling Its Complexities, Diagnostic Challenges, and Multifaceted Treatment Approaches

**DOI:** 10.7759/cureus.61854

**Published:** 2024-06-06

**Authors:** José Pablo Zárate-García, Luis Alberto Ortega-Porcayo, Maria Fernanda Tejada-Pineda, Júlia Moscardini-Martelli, Samuel Romano-Feinholz, Juan Antonio Ponce-Gómez, Marcela Amparo Osuna-Zazueta, Alexa Natalia Zárate-García, Mariana Elisa Guillén-Camacho, Sergio M Jiménez

**Affiliations:** 1 Department of Neurosurgery, American British Cowdray (ABC) Medical Center, Mexico City, MEX; 2 School of Medicine and Health Sciences, Tecnológico de Monterrey, Monterrey, MEX; 3 Department of Neurosurgery, Instituto Nacional de Neurología y Neurocirugía Manuel Velasco Suárez, Mexico City, MEX; 4 Department of Neuroanesthesiology, American British Cowdray (ABC) Medical Center, Mexico City, MEX; 5 School of Medicine and Health Sciences, Facultad Mexicana de Medicina Universidad La Salle, Mexico City, MEX; 6 Radiosurgery Unit, Instituto Nacional de Neurología y Neurocirugía Manuel Velasco Suarez, Mexico City, MEX; 7 Department of Neurosurgery-Radiosurgery, American British Cowdray (ABC) Medical Center, Mexico City, MEX

**Keywords:** trigeminal nerve biopsy, cranial nerve enhancement, multiple cranial nerve neuropathy, cranial nerve involvement, perineural invasion (pni), cutaneous squamous cell carcinoma (scc)

## Abstract

Cutaneous squamous cell carcinoma is the second most common neoplasm among non-melanoma skin cancers. When associated with perineural invasion of the cranial nerves, with clinical features often observed in trigeminal and facial nerves due to their cutaneous extension, it may lead to a worse prognosis. This paper introduces a rare case of an 81-year-old male, with a history of a moderately differentiated invasive carcinoma of the left frontal region with perineural invasion on the left trigeminal cranial nerve. The case underscores the aggressive nature of the intraneural infiltration by squamous cell carcinoma and the challenges in managing such advanced malignancies.

## Introduction

Cutaneous squamous cell carcinoma (CSCC) is the second most common non-melanoma keratinocyte carcinoma [1]. It accounts for 20% of all cutaneous malignancies and its incidence ranges from five to 499 per 100,000 patients, and this incidence is likely to increase due to the rising elderly population worldwide and better skin cancer detection [2].

Risk factors for developing CSCC include exposure to ultraviolet radiation, increasing age, fair skin, male gender, smoking, infection with β‐human papillomaviruses (HPV), and immunosuppression [3]. Case presentation of CSCC associated with perineural invasion (PNI), where malignant cells infiltrate nerve sheaths [4], results in a more aggressive clinical course and management complexity.

We present a rare case of an 81-year-old male with perineural extension and invasion to the trigeminal nerve from a CSCC of the left frontal region and a brief literature review on perineural invasion.

## Case presentation

An 81-year-old man presenting with a localized neurological deficit was admitted to the emergency department in May 2023. The patient had a history of a moderately differentiated CSCC located in the left frontal region (25x20x5 mm approximately) managed via surgical resection (February 2021); the pathology report described free microscopic resection margins, with no lymphatic, vascular, or perineural invasion (total surgical resection area 45x3x6 mm). Radiological follow-up with FDG-18 positron emission tomography (PET) scan at 20 months after surgery showed no active disease or progression.

**Figure 1:**
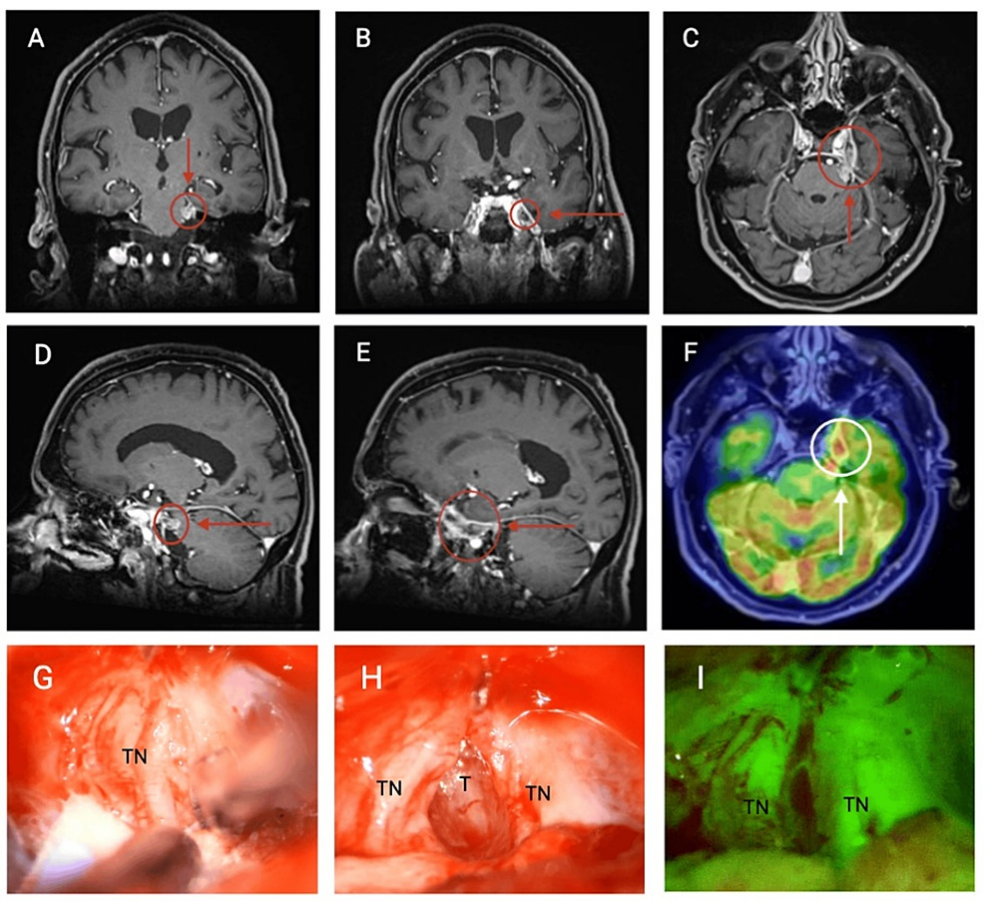
Trigeminal nerve invasion. MRI with gadolinium in coronal (A, B), axial (C), and sagittal (D, E) planes showing an inflammatory process that involves the trajectory of the left trigeminal nerve with extension towards the ipsilateral cavernous sinus, which shows continuity through the superior orbital fissure through the ophthalmic branch of the trigeminal cranial nerve (V1), oculomotor nerve (third cranial nerve), and trochlear nerve (fourth cranial nerve) as well as the mandibular and maxillary branch (V2, V3) in the trajectory of foramen ovale and rotundum. 18-FDG PET/CT scan in the axial plane (F) showed a hypermetabolic lesion in the left cavernous sinus involving the left trigeminal trajectory (6.9 SUVmax). A retrosigmoid craniotomy showed the left trigeminal nerve (G) with perineural tumor invasion positive to fluorescein (I).  The nerve was dissected and opened to take a core central biopsy of the tumor (H). PET: positron emission tomography

Upon admission, he had left ptosis, external ophthalmoplegia, absent light and consensual pupillary reflex, left hypoesthesia on V1 and V2, and left peripheral facial nerve palsy. Cavernous sinus syndrome was diagnosed, and a brain MRI showed an infiltrative tumor involving the trigeminal nerve with extension towards the cavernous sinus (Figure 1). Biochemical testing on the cerebrospinal fluid (CSF) showed proteinorachia and pleocytosis (glucose 51.6 mg/dL, leucocytes 3 cells/mm^3^, polymorphonuclear cells 33% and mononuclear cells 67%, proteins 80.9 mg/dL, and DHL 19 U/L); CSF cultures and polymerase chain reaction were negative for microbiological agents. FDG-18 PET/CT scan showed generalized cortical hypometabolism and a focal hypermetabolic lesion in the left cavernous sinus (Figure 1).

In June 2023, a left trigeminal nerve biopsy was performed using a retrosigmoid craniotomy (Figure 1). Pathology reported intraneural infiltration by a moderately differentiated keratinizing squamous cell carcinoma (p40 positive, p63 positive, p16 negative, s100 negative, SOX10 negative, ki67 25%), findings consistent with metastatic process from known primary. Although adjuvant radiotherapy and immunotherapy were proposed, the patient did not receive further treatment, and unfortunately, he succumbed to disease progression.

## Discussion

Skin cancer is one of the most common neoplasms, with 90% of cases occurring in the head and neck regions, due to sun exposure. Basal cell carcinoma is the most common malignancy in this cluster, followed by CSCC, which is known to spread to regional lymph nodes (15%) and PNI (2-15%) [5,6]. Areas of common metastasis of CSCC include the lung, liver, bone, kidney, adrenals, and pleura; brain metastasis seldom occurs in head and neck CSCC [7]. Advanced CSCC is locally unresectable and invasive (affecting bone, nerve, or muscles), generates distant metastases, and requires systemic treatment [8].

PNI is the infiltration and spread of neoplastic cells along the nerve sheath [4,9]. Nerves have three layers called the endoneurium, perineurium, and epineurium, which is a dense irregular connective tissue with high selective permeability [10]. The tumor disseminates in non-contiguous regions along the nerve sheath through the endoneurium or perineurium [3]. It initiates in a centripetal direction from the peripheral extension, but it may change into an inverse direction [11]. As the metastasis extends, there may be areas of the nerve that are uninvolved or involved microscopically disabling to see the full extension of the disease [11], for which clear surgical margins may not be sufficient for the treatment of PNI [3]. Perineural extension inside the skull is divided into three zones: zone 1, peripheral; zone 2, central/skull base; and zone 3, cisternal [12]. It most commonly involves trigeminal and facial nerves; still, it can affect any cranial nerve in contact with mucosal and cutaneous surfaces or adjacent to a minor or major gland [10,11]. The spread of the tumor is foreseeable knowing the anatomical patterns of the region of the primary tumor. For example, tumors of the forehead, eye, and ethmoid and frontal sinuses may extend along V1, and tumors of the parotid gland can go along the auriculotemporal branch of V3 or the facial nerve, being misdiagnosed as trigeminal neuralgia or Bell's palsy [3].

The first case of primary cutaneous carcinoma with perineural invasion was described in 1862 by Neumann, in a patient with primary carcinoma of the lower lip which spread along the mental nerve [13]. The incidence of PNI in CSCC varies substantially, being higher in head and neck carcinoma in comparison with other areas, with a range from 2% to 14% [2,3]. Neuropathy is observed in 30-40% of PNI cases and is related to lower survival rates, increased rates of recurrences, and a greater risk of metastasis to adjacent structures, distant sites, and lymph nodes [3]. Patients with PNI had a 16% survival rate up to five years, in comparison with a 44% survival rate without PNI; 46% of patients with CSCC and PNI had died within two years or had recurrence versus 9.1% of patients without PNI [13].

The American Joint Committee on Cancer (AJCC) included the following high-risk characteristics of squamous cell carcinoma: Clark level IV, invasion >2 mm, PNI, location on the ear or vermilion lip, and poorly differentiated or undifferentiated growth pattern. The National Comprehensive Cancer Network (NCCN) includes as high-risk characteristics all head and neck carcinomas [4,8]. Risk factors for poor prognosis, recurrence, and metastatic disease of CSCC are lesions >2 cm in diameter, >2 mm tumor thickness, poor differentiation, hypodermis invasion, previous radiation, and perineural invasion [14]. Desmoplasia and increasing tumor thickness are the most relevant prognostic factors of recurrence and poor prognosis [15,16]. Patients with immunosuppression develop higher rates of desmoplastic CSCC and PNI [15]. 

The mechanism of PNI is not well defined; thus, it has been researched that neural cell adhesion molecules, such as the expression of TrK-A (tropomyosin receptor kinase A), a high-affinity receptor for the nerve growth factor which participates in the maintenance of the central and peripheral nervous system, is almost double in head and neck CSCC compared to non-head and neck areas, but it lacks statistical correlation between TrK-A expression and PNI in CSCC [9,13,17]. It has been considered that the tumor cells interact with the nerve microenvironment as they spread centripetally with skip lesions. It also secretes molecules that promote neurite growth, which includes brain-derived neurotrophic factor (BDNF), nerve growth factor (NGF), neurotrophin-3 (NT-3), neurotrophin-4 (NT-4), neural cell adhesion molecule (NCAM), substance P, glial cell line-derived neurotrophic factor (GDNF), and higher rates of TrkB (affinity receptor for BDNF and NT-4), among others [10,17,18].

PNI is unpredictable and difficult to treat. Several patients are misdiagnosed, which increases disease progression [19]. Patients with clinical perineural invasion have a greater risk of recurrence and metastasis and low survival rates than asymptomatic perineural invasion [14]. Bilateral cranial nerve involvement is rare, as well as leptomeningeal metastases, but they should be considered a differential diagnosis [11,19]. The diagnosis is made clinically, radiologically, and histopathologically. Clinical features are most frequently in the trigeminal and facial cranial nerves due to their cutaneous distribution. Histological evaluation can include NGFR (nerve growth factor receptor) and S-100 staining [8,14]. Radiologically findings can be made through MRI or CT, showing atypical enhancement, obliteration of the normal trajectory of the nerve, and erosion of adjacent structures [13,14,20]. A high-resolution gadolinium MRI is preferred, which demonstrates nerve enhancement and has a 95% sensitivity and an 84% specificity detecting PNI [13,21]. CT can be used additionally in cortical bone involvement. PET-CT scan is an invaluable tool for detecting primary unknown tumors, as well as being useful in the evaluation and follow-up assessment, but the sensitivity of PET/CT scans with FDG is reported to have low detection for perineural invasion [11]. Nerve biopsies are suggested for the definitive diagnosis of perineural invasion and should be considered in the presence of clinical manifestations and PNI risk factors (prior neoplastic skin lesion, old age males, >2 cm primary lesion, >2 mm thickness of primary lesion involving hypodermis, etc.) [21], even if the neuroimaging is negative.

Radical excision is necessary for the treatment of PNI. Mohs micrographic surgery, named after his founder Frederic E. Mohs, is the most accepted treatment in CSCC with PNI, as it attempts better surgical margins and has better rates of survival outcomes and lower recurrence in comparison to other surgical modalities [13]. It also seems to have greater sensitivity to detect tumor spread, making it the best option as it has greater control rates than standard excision [14]. But even when treated with Mohs surgery, 5% of CSCC will metastasize [20]. Adjuvant radiotherapy followed by the surgical resection of CSCC may be considered with positive surgical margins or extensive tumor infiltration [13,22]. Postoperative radiotherapy has been shown to improve local control and overall survival after surgical resection in patients with PNI and cavernous sinus involvement [5]. Patients with recurrent lesions, mid-face locations, and proximity to the facial and trigeminal cranial nerves should also be considered to receive adjuvant radiotherapy. Immunocompromised patients have higher incidence rates of aggressive neoplasms and should receive adjuvant radiotherapy as well [16].

In case of PNI <0.1 mm and negative surgical margins, clinical follow-up is recommended. For patients with PNI >0.1 mm and negative surgical margins, adjuvant radiotherapy is indicated. When the disease is affecting zone 3 of perineural extension, palliative radiotherapy is indicated with low cure possibilities. Zone 3 surgical procedures have a high risk of neural and vascular lesions due to anatomical complexity [23]. Cemiplimab and pembrolizumab are two immunotherapy-approved options with locally advanced, recurrent, or metastatic CSCC. Both are human monoclonal antibodies specific for programmed death receptor-1 [8].

Focal cranial nerve invasion and microscopic PNI have a better prognosis compared to extensive invasion, clinically symptomatic PNI, or radiographic findings of PNI [20]. All recurrences present in cases with positive surgical margins, whereas cases with extensive PNI (clinical symptoms or imaging findings) had 45% more risk of recurrence even with clear surgical margins and 50% more risk of local recurrence with >0.1 mm in PNI diameter. Patients with PNI <0.1 mm in diameter had better outcomes and less risk of local recurrence, regional metastasis, distant metastasis, and disease-specific mortality [22].

## Conclusions

The high incidence of CSCC in the head and neck region, coupled with the potential for PNI, emphasizes the importance of early detection and heightened suspicion of this disease among healthcare professionals. Its unpredictable nature, its association with neuropathy, and the difficulty in achieving clear surgical margins underscore the need for comprehensive clinical, radiological, and histopathological evaluation. We presented a case report of an 81-year-old patient with high-risk factors for relapse or metastatic disease from CSCC. The article underlines the importance of recognizing high-risk features in CSCC, as they are crucial for patients' survival outcomes.
